# Systematic Review With Meta-Analysis: Are Muscle Transfers a Satisfactory Treatment Option to Restore Shoulder Abduction in Delayed Adult Brachial Plexus Injuries?

**DOI:** 10.7759/cureus.12914

**Published:** 2021-01-26

**Authors:** Shady Hermena, Ali Assaf, Oliver Donaldson

**Affiliations:** 1 Trauma and Orthopaedic Department, Yeovil District Hospital NHS Foundation Trust, Yeovil, GBR; 2 Trauma and Orthopaedic Department, Isle of Wight NHS Trust, Isle of Wight, GBR

**Keywords:** shoulder abduction, muscle transfer, brachial plexus injury

## Abstract

Brachial plexus injuries usually result in significant upper limb disabilities and shoulder joint instability. Primary nerve reconstruction procedures are more effective if performed within six months from the injury. Secondary procedures, including muscle transfers, are usually indicated for delayed presentation (>6 months) or when the outcomes of primary procedures are unsatisfactory.

A comprehensive systematic search of the MEDLINE, EMBASE, AMED, PubMed, and Cochrane databases was conducted in line with the Preferred Reporting Items for Systematic Reviews and Meta-Analyses (PRISMA) guidelines. Data, including demographic information, time to surgery, the extent of brachial plexus injury, surgical techniques, follow-up duration, and functional outcomes were collected and tabulated. Meta-analysis was conducted using Review Manager (RevMan) 5.4 software ([Computer program]. Version 5.3. Copenhagen: The Nordic Cochrane Centre, The Cochrane Collaboration, 2014). Seven studies were eligible to be included in this review, with a total of 218 patients. The average patient age was 28.39 ± 3 years, with a mean time to surgery of 29.87 ± 18 months. Forty-six (46) patients (21.10%) were treated as delayed presentation and 172 patients (78.89%) had muscle transfer performed as a secondary procedure. The mean time at follow-up was 18.86 ± 13.5 months. Upper trapezius muscle transfer was the most common transferred muscle (100%) either in isolation (n=159, 72.93%) or in combination with lower trapezius transfer (n=59, 27.06%). The mean preoperative and postoperative shoulder abduction were 12.22 ± 10.09 degrees and 58.36 ± 32.33 degrees, respectively (p < 0.05). Meta-analysis shows a statistically significant difference (CI at 95%, p<0.05) favoring postoperative shoulder abduction.

Muscle transfers especially upper trapezius transfer could be a satisfactory secondary procedure to restore shoulder abduction and enhance shoulder joint stability.

## Introduction and background

The brachial plexus is a neural network that originates from the lower four cervical and first thoracic nerve roots, providing motor and sensory innervation to the upper limb [1]. Adult traumatic brachial plexus injuries (ATBPI) usually result in significant upper limb motor and sensory dysfunction. These injuries result from various modes of closed trauma, such as nerve traction or rupture, and, less commonly, from open lacerations and gunshots [2]. Brachial plexus injuries are usually classified as the upper trunk (C5, C6+/- C7), lower trunk (C8, T1), or pan plexus injuries (C5-T1) [3]. Millesi classified brachial plexus injuries into preganglionic, postganglionic, trunk, or cord injuries [4]. Another classification describes the injury relative to the clavicle, thus supra-clavicular, retro-clavicular, and infra-clavicular [3]. Loss of shoulder abduction usually results from C5, C6+/-C7 lesions due to paralysis of the deltoid and supraspinatus muscles [5]. Although it is difficult to ascertain the exact annual incidence of ATBPI, many studies suggested an increasing incidence of ATBPI as a result of increasing motor vehicle accidents [5-6]. Male patients aged between 15 and 25 years old are the most affected cohort [6].

It is essential to understand the anatomy and kinematics of shoulder abduction to be able to restore this function [7]. The shoulder complex involves three physiological joints and one articulation: glenohumeral joint, acromioclavicular joint, sternoclavicular joint, and scapulothoracic articulation [8]. A stable shoulder joint is required for effective distal elbow and hand function [9]. Static glenohumeral stabilizers incorporate the glenoid labrum and capsuloligamentous components. Dynamic stabilizers of the shoulder complex consist of the periscapular muscles in addition to the rotator cuff muscles: supraspinatus, infraspinatus, subscapularis, and teres minor [8]. Shoulder movements require synergistic mechanisms of the muscles' co-contractions, appropriate scapula positioning, and coordination of the glenohumeral and scapulothoracic articulations, which is referred to as scapulohumeral rhythm [10]. Full active shoulder abduction necessitates the simultaneous action of two muscle groups: the muscle acting around the scapulothoracic articulation and those around the glenohumeral joint [11]. The supraspinatus muscle is accountable for the first 15 degrees of abduction, whereas the deltoid muscle is responsible for the abduction of the arm from 15 to 90 degrees [12]. During shoulder abduction, the deltoid muscle contracts to create a vertical shear force, which is opposed by the combined action of rotator cuff muscles to keep the humeral head congruent and minimize translation [10]. Therefore, inferior subluxation is a common finding in brachial plexus injuries due to deltoid paralysis. Simultaneous action of the serratus anterior and trapezius muscles is required to rotate the scapula to accommodate full shoulder abduction [12].

Restoration of shoulder abduction permits a more comprehensive functional range of movement of the upper limb and higher satisfaction in performing daily activities [13-14] and is, therefore, a primary goal in managing patients with ATBPI.

The time interval between the injury and the surgical intervention is one of the main factors that guide the therapeutic algorithm [5,15]. The best functional outcomes can be achieved via primary nerve reconstruction comprising direct nerve repair, neurolysis for scar compression, nerve grafting, and nerve transfers performed no later than six months following injury [16-17]. As a result of interruption of the motor innervation, a time-dependent irreversible degeneration commences at the motor endplate [5,15]. Reinnervation procedures performed 18-24 months following injury usually result in a functionless muscle [18-19]. Mainstream surgical therapy, therefore, advises that, if the patient presents within six months of injury, reinnervation procedures should be considered first due to the favorable results compared to muscle transfers [20-22].

Muscle transfers to improve shoulder abduction are usually indicated as secondary procedures due to poor outcomes from primary reinnervation or delayed presentation [17,23]. The decision about secondary reconstructive procedure depends on the type of ATBPI, satisfactory passive shoulder range of movement, as well as the power and availability of the local muscles [7,12,24-25]. The selected donor muscle for transfer must have at least grade 4 power according to the Medical Research Council (MRC) Grading scale, as there is an expected drop in one grade in muscle power following tendon transfer [7,12,24-25]. Various muscles have been transferred to the proximal humerus to restore shoulder abduction [26-33].

Although there have been previous meta-analyses investigating nerve reconstruction outcomes, there are no systematic reviews of the available literature investigating the outcomes for muscle transfer procedures. This study aimed to perform a systematic review with a meta-analysis of the published studies evaluating the outcomes of muscle transfers to restore shoulder abduction following a delayed presentation of ATBPI or poor outcome from a primary reinnervation procedure.

The primary outcomes measured in this review were: the range of recovered shoulder abduction, improvement in the disabilities of the arm, shoulder, and hand (DASH) score, and change in shoulder stability after muscle transfers for delayed ATBPI as defined by the ability to maintain the humeral head in the glenoid fossa [34]. Shoulder instability could occur anteriorly, posteriorly, or in multiple directions. Instability is commonly subjectively reported due to the interfere with daily activates [35]. Objectively, instability could be detected by clinical tests, including load and shift, drawer, apprehension and drawer, or even radiologically [36].

## Review

Methods

This study was conducted in line with the methodology of the systematic review presented in the Cochrane Systematic Reviews handbook, and it is reported according to Preferred Reporting Items for Systematic Review and Meta-Analysis (PRISMA) guidelines.

Search Strategy

A comprehensive systematic search of the literature was conducted using the electronic databases: MEDLINE, Embase, Allied and Complementary Medicine Database (AMED), PubMed, and Cochrane. The search strategy included a set of terms for "muscle transfer", "shoulder abduction", and "brachial plexus injury", which were then connected by the Boolean operator. The search results were supplemented by a manual search of relevant reviews and their references to ensure all eligible studies were ultimately included.

Inclusion Criteria 

Published articles between 1940 and up to May 2020, which met the following criteria, were included:

1. Randomized controlled studies, controlled trials, or cohort studies reporting the outcomes of muscle transfers to restore shoulder abduction for brachial plexus injuries in the adult population

2. The study includes details of surgical intervention, follow-up duration, and functional assessment details of preintervention and postintervention recovered shoulder abduction

3. Articles published in English

*Exclusion Criteria* 

Studies reporting muscle transfer as an intervention for poliomyelitis, rotator cuff tears, or obstetric brachial plexus lesions were excluded. Studies published in languages other than English were also excluded.

Screening and Assessment of Eligibility

Articles were initially included based on their title and abstract. Duplicate studies were excluded. The full-text review then was conducted for confirmation of inclusion.

Data Extraction and Quality Assessment

The following data were extracted: demographics (age and sex), the time interval between the injury and surgical intervention, the extent and site of brachial plexus injury, surgical techniques, including choice of the donor muscle, length of follow-up, functional outcomes, including a restored range of movement and patient-rated outcome measures, and DASH score. The systematic search did not reveal any relevant randomized controlled study or controlled studies. Quality assessment for cohort studies was performed using the Methodological Index for all Non-Randomized Studies (MINORS) score by two independent reviewers [37].

Data Analysis

Microsoft Excel (Microsoft Corporation, Redmond, WA) was used for data collection and statistical analysis. Each study was reviewed by two reviewers and data were incorporated. Review Manager (RevMan) 5.4 software ([Computer program]. Version 5.3. Copenhagen: The Nordic Cochrane Centre, The Cochrane Collaboration, 2014) was used for the meta-analysis [38].

Results

Study Selection and Quality Assessment 

**Figure 1:**
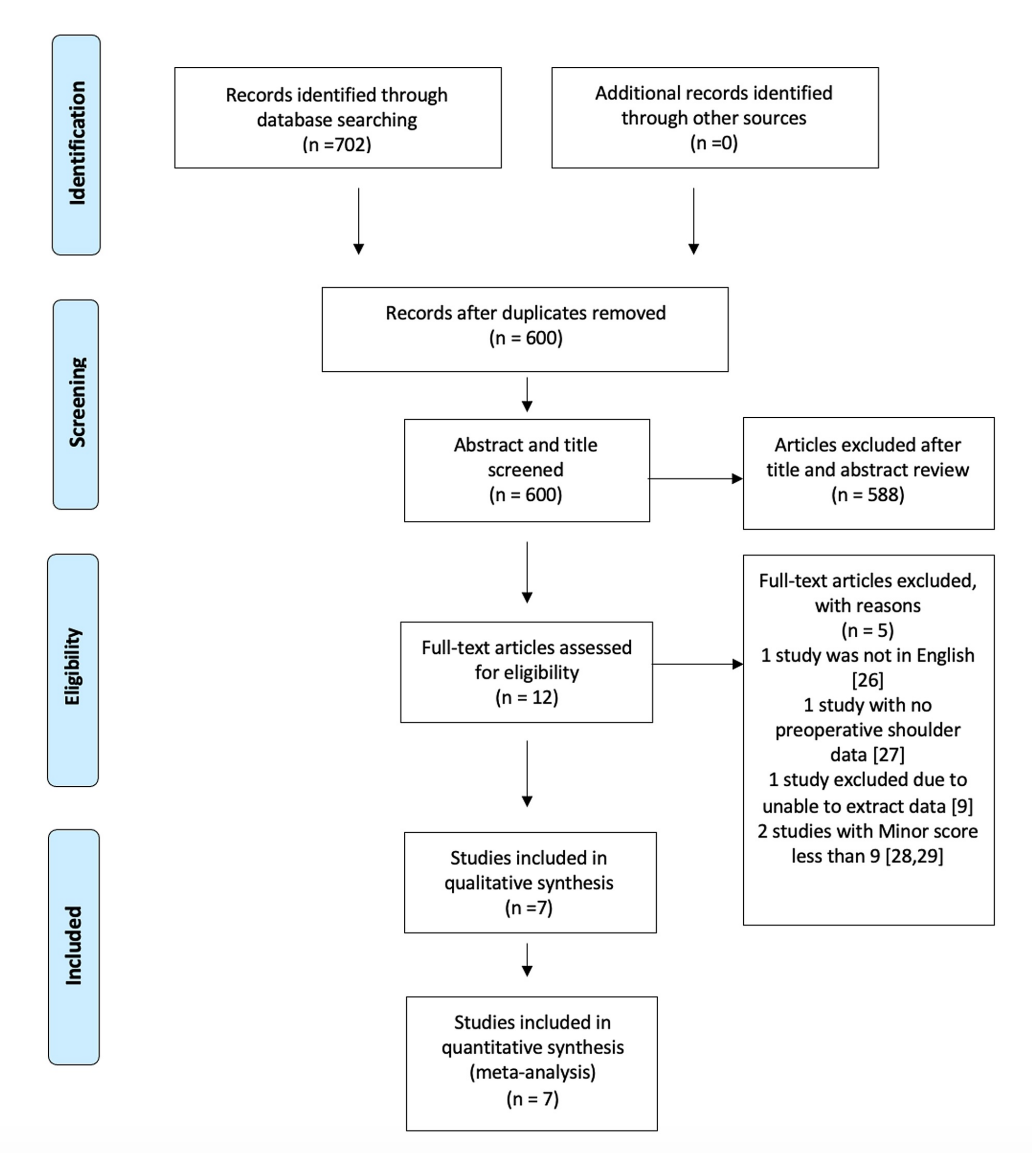
PRISMA flow of the screening process PRISMA: Preferred Reporting Items for Systematic Review and Meta-Analysis

Figure 1 represents the screening process. The extensive primary literature search revealed 702 articles with no studies identified from other resources. One hundred and two records were removed due to duplication. After title and abstract review, a further 588 studies were excluded. A full-text review for the remaining 12 articles was performed. No randomized nor blinded studies were identified. Five studies were not eligible for inclusion. Altmann et al.'s study (2006) was not in English [26]. Kotwal 1990 et al. was excluded, as there was not enough pre and post-intervention data available [27]. Two studies scored less than 9 according to the MINOR quality score and were also excluded [28-29]. One study that reported multiple transfers around the shoulder and elbow was excluded, as data extraction for shoulder abduction treatment was not possible [9].

Included Studies 

The systematic screening revealed seven eligible studies: five cohort studies and two case series with a total of 218 patients (Table 1). The average patient age was 28.39 ± 3 years, with a mean time to surgery of 29.87 ± 18 months. The mean time at follow-up was 18.86 ± 13.5 months. Forty-six patients (21.10%) were treated as delayed presentation and 172 patients (78.89%) had muscle transfer performed as a secondary procedure following a poor outcome from primary innervation procedures.

**Table 1:** Shoulder abduction before and after tendon transfer surgery for ATBPI N, number of patients; t, mean time elapsed from injury to surgical treatment in months; SD, Standard Deviation; Pre-op, preoperative; Post-op, Postoperative; U, upper trunk; L, lower trunk; P, pan plexus; NR, not reported; ATBPI, Adult traumatic brachial plexus injuries

Study	N	Age, years	Brachial plexus injury	t, months	Donor muscle	Follow-up, months	Abduction ( °) mean (range) or SD		T test for shoulder abduction
							Pre-op	Post-op	
Karki 2020 [39]	12	27	U = 5 Pan = 7	24	Upper Trapezius	6	37 (10-90)	116 (10-180)	P < 0.05
Agrawal 2015 [40]	32	23.5	NR	≥ 24	Upper Trapezius	8.25	7.5 (0-30)	85 (45-145)	NR
Elhassan 2012 [30]	52	27	U = 34 L = 5 Pan = 13	≥ 12	Combined Upper and Lower Trapezius	19	10 (0-20)	60 (40-100)	P < 0.05
Bertelli 2011 [31]	7	27.57	U = 7	20.85	Combined Upper and Lower Trapezius	11.7	0 ± 0	Increase of 37	NR
Singh 2007 [41]	8	31.5	U = 7 Pan = 1	20.3	Upper Trapezius	15.3	13.7 (0-30)	116 (45-180)	P < 0.05
Rühmann 2005 [32]	80	31	NR	72	Upper Trapezius	28.8	6 (0-45)	34 (5-90)	NR
Aziz 1990 [33]	27	31.2	U = 22 Pan = 5	31.3	Upper Trapezius	14.6	3.5 (0-30)	45.4 (20-120)	P < 0.05

Transferred Muscles

Upper trapezius muscle transfer was the most common procedure seen in all of the patients, either as a sole procedure (n=159, 72.93%) or in combination with lower trapezius transfer (n=59, 27.06%).

Extent of Brachial Plexus Injury and Transferred Muscles

Only five of the included studies reported on the type of brachial plexus injury, described in Table 1. Of the patients with upper plexus injury (n=75, 34.40%), 34 (48.57%) had had upper trapezius transfer, whereas the other 41 patients (54.66%) had a combined upper and lower trapezius muscle transfer. The five patients (2.29%) that had sustained an isolated lower trunk injury underwent a combined upper and lower trapezius muscles transfer. For the patients that had pan plexus injury (n=26, 11.93%), exactly half underwent upper trapezius transfer and the other half had a combined upper and lower trapezius muscle transfer. The remaining 112 patients (51.38%) did not have a description of their injury, and all underwent upper trapezius transfer.

Upper Trapezius Transfer

Upper trapezius transfer was the most common muscle transfer performed but there was no consistency between surgical technique or fixation methods between studies. Karki et al. (2020) transferred a combination of the trapezius muscle tendinous unit and tensor fascia lata strip to the deltoid muscle insertion in 12 patients (4.68%) [39]. Karki et al. (2020) sutured the triple folded harvested fascia lata to the trapezius musculotendinous unit then sutured the combined unit over the anterolateral aspect of the deltoid muscle close to its insertion with the shoulder abducted 110 degrees [39].

Aziz et al. (1990) transferred the osteotomized lateral 1 cm of the clavicle and the acromion with their trapezius insertion to the proximal humerus in 27 patients (10.54%). Aziz 1990 et al. fixed the transferred acromioclavicular fragment to the humerus with two 4.5 mm cortical screws ensuring firm bone-to-bone contact [33]. Bertelli (2011) and Ruhmann et al. (2005) transferred osteotomized acromion fragment with its trapezius attachment to the proximal humerus in 87 patients (33.98%). Bertelli (2011) used one screw and washer to fix the transferred bony fragment while Ruhmann (2005) et al. used two 6.5 cancellous screws [31-32]. Agrawal (2015) transferred a clavicular bony fragment with its trapezius insertion to the humerus and fixed it with two 4 mm cancellous screws in 32 patients (12.5%) [40]. Singh (2007) et al. dissected and detached the trapezius insertion along with the periosteum and sutured it to the insertion of the deltoid muscle in eight patients ( 3.12%) [41].

Dual Upper and Lower Trapezius Transfer

Two studies reported the outcomes of dual upper and lower trapezius transfer (n= 59, 27.06%). Elhassan transferred the tendinous insertion of the lower trapezius lengthened by an Achilles tendon allograft to the infraspinatus muscle insertion and the osteotomised acromion with the attached upper trapezius to the proximal humerus distal to the greater tuberosity. Elhassan anchored the transferred acromion with two or three partially threaded 3.5-mm cortical screws with washers with the shoulder in 80 degrees of abduction [30]. Bertelli fixed the transferred osteotomised acromion with a screw and washer to the proximal humerus and sutured the deltoid muscle over while the shoulder was abducted to 90 degrees. In the same setting, Bertelli sutured the lower trapezius tendon after dissecting the fascial attachments over the spine of the scapula to the infraspinatus tendon under maximal tension, with the shoulder maximally externally rotated [31].

Range of Recovered Shoulder Abduction 

All studies detailed enhancement in shoulder abduction post muscles transfer. The mean preoperative and postoperative shoulder abduction were 12.22 ± 10.09 degrees and 58.36 ± 32.33 degrees, respectively (p < 0.05) with a mean gain of 46 degrees. Those patients who underwent a combined upper and lower trapezius transfer had a mean gain in shoulder abduction of 56.92 ± 26.47 degrees, whereas those who underwent exclusively upper trapezius transfer had a mean postoperative shoulder abduction gain of 56.44 ± 29.16 degrees (p = 0.648).

Patient Self-Reported Outcomes

The disabilities of the arm, shoulder, and hand (DASH) outcome questionnaire is a widely accepted measurement tool for self-rated upper extremity disability and symptoms [42-43]. The DASH score was first introduced by the American Academy of Orthopaedic Surgeons in collaboration with other organizations [44]. The DASH consists of 21 disability and symptom items, scored from 0 (no disability) to 100 (most severe disability) [42-44]. According to the study conducted by Gummesson et al. evaluating the longitudinal construct validity of the DASH score, a 10-point difference in mean DASH score is considered the minimal important difference [42]. Two studies, Elhassan et al. and Karki et al., reported the outcomes of the preoperative and postoperative DASH score [30,39]. Elhassan et al. included 52 patients (20.31%) and reported an average drop in DASH score of 12 points following dual upper and lower trapezius transfer. Karki et al. involved 12 patients (4.68%) and reported an average drop in the DASH score of 47 following upper trapezius transfer [39].

Shoulder Stability 

Four studies (n=167 patients, 60%) reported an increase in shoulder stability after muscle transfers. There was no consistency between studies in how shoulder instability was assessed, as some assessed stability subjectively and clinically (Elhassan and Singh), others clinically and radiologically (Rühmann), and others did not specify how it was assessed (Aziz). Both Elhassan and Aziz (n=79, 36.24%) reported 0% postoperative instability (p < 0.05). Singh reported that all patients (n=8, 3.67%) improved (p < 0.05), however, no quantitative assessment was provided. Rühmann reported an 18% postoperative instability incidence in their cohort (n=80, 36.70%). Of the 167 patients that had postoperative shoulder stability reported, 115 (68.86%) received upper trapezius transfer with postoperative instability ranging from 0-18%, whereas 52 (23.85%) received a combined upper and lower trapezius transfer with 0% of shoulder instability reported. Statistical analysis was unable to be performed due to the lack of consistency between the stability reporting methods.

Meta-Analysis of Preoperative and Postoperative Shoulder Abduction

**Figure 2:**
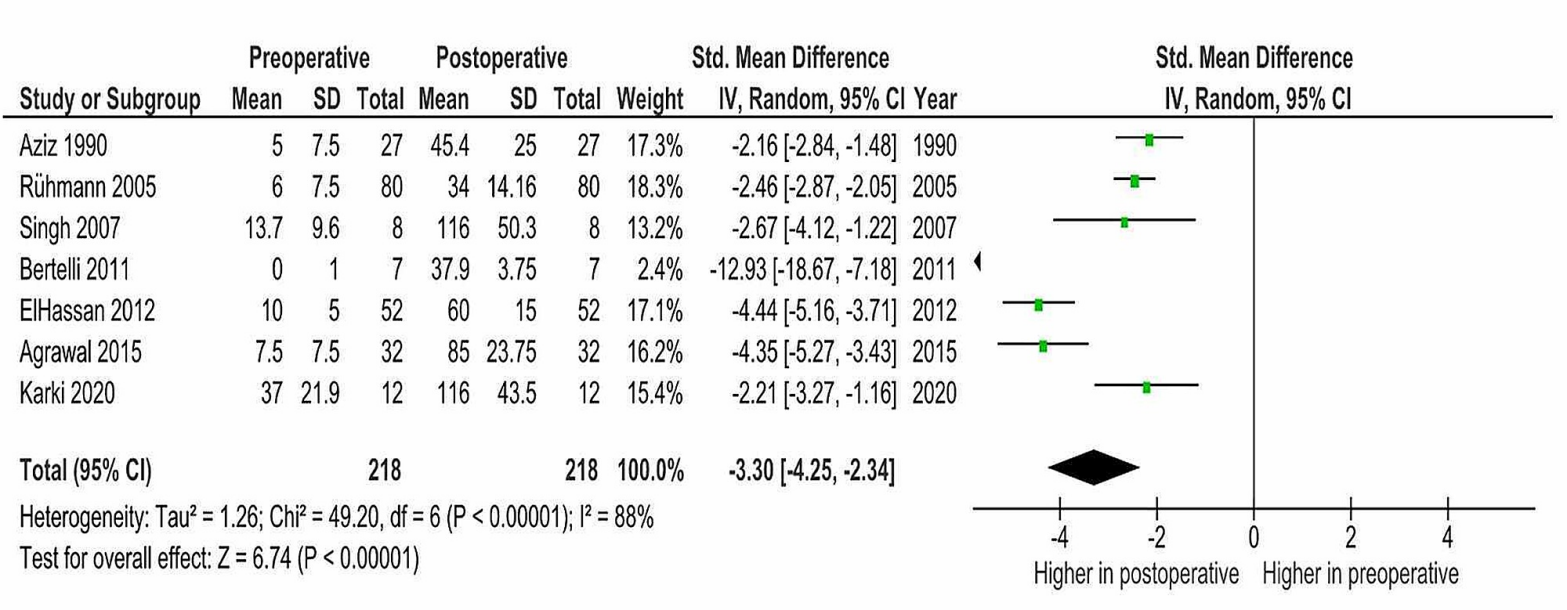
Meta-analysis of preoperative and postoperative shoulder abduction

A meta-analysis was carried out on preoperative and postoperative shoulder abduction. For the studies that had not reported the standard deviation (SD), and this was not able to be calculated from the available data (Agrawal 2015, Elhassan 2012 and Ruhmann 2005 et al.), SD was estimated using the estimation system proposed by Xiang Wan et al. using sample size and range [45]. Due to the high heterogeneity of the studies (I2 = 89%), an inverse variance with the random-effects model was used as an analysis method. The analysis was carried out using RevMan 5.4 [38]. As illustrated in Figure 2, the meta-analysis shows a statistically significant difference (CI at 95%, p<0.05) favoring postoperative shoulder abduction.

Discussion

Upper Trapezius Transfer 

Isolated upper trapezius muscle transfer was the most common muscle transfer to restore shoulder abduction, representing 72.93 %of the involved patients in this review. The common use of the trapezius muscle as a donor muscle for transfer could be explained by trapezius muscle-sparing following brachial plexus palsy, as it is innervated by the spinal accessory nerve with contributions from C3 and C4. Trapezius muscle hypertrophy due to the overuse following deltoid muscle paralysis is another cause favoring its use for transfer [46-47]. However, the spinal accessory nerve is commonly used as a donor nerve for nerve reconstruction procedures, resulting in paralyzed middle and lower trapezius but sparing the upper trapezius [30,48]. Therefore, the lower trapezius muscle is less available for transfer compared to the upper trapezius muscle.

There are various techniques for trapezius transfer described in the literature. The highest range of recovered shoulder abduction (average 116 degrees ) was achieved by Karki et al. (2020) and Singh et al. 2007, which includes 20 patients in total and so their results must be interpreted with caution [39,41]. Karki et al. (2020) used a technique similar to the original Mayer's technique (1927) where they extended the trapezius with a folded tensor fascia Lata graft and attached it as distally possible to the deltoid insertion [39,49]. Singh et al. (2007) dissected and detached the trapezius from its insertion along with the periosteum and sutured it to the insertion of the Deltoid muscle [41]. Both Karki et al. (2020) and Singh et al. (2007) reported improvement in shoulder stability post-transfer. Karki et al. (2020) reported a post-transfer reduction in DASH score of 47 points while Singh 2007 et al. did not report DASH scores [39,41].

Trapezius muscle transfer was first described over a century ago by Hoffman in 1891. Further techniques were described by Lewis and Lange in 1910 and 1911, respectively. Bateman (1955) further modified trapezius transfer by osteotomising the trapezius insertion from the acromion and transferring it to the proximal humerus. The main limitation of this technique was the short lever arm. Saha (1967), consequently, modified this technique by mobilizing the upper and middle segments of the trapezius muscle from its origin and thus transfer was made 5 cm longer. Therefore, Saha’s technique allowed the longer lever arm by fixing the acromion to the humerus just below the greater tuberosity with two 6.5 mm cancellous screws with the arm in 80 to 90 degrees of abduction [48,50]. Saha’s technique was adopted by Ruhmann et al. (2005) and Bertelli 2011 [31-33]. The study has demonstrated a number of surgical techniques used to perform upper trapezius transfers with a number of different fixation methods. This study was unable to conclude which technique or fixation method was best.

Combined Upper and Lower Trapezius Transfer

Lower trapezius transfers have been used to primarily improve shoulder external rotation [30,48]. However, dual upper and lower trapezius transfer has been found to improve shoulder abduction biomechanics as well as shoulder external rotation [30,48 ]. It has been argued that upper trapezius transfers only provide a tenodesis effect on the glenohumeral joint and the recovered abduction was mainly achieved by the active action of periscapular muscles, pectoralis major, biceps, and triceps muscles [50]. This could be explained by the upward humeral head migration effect following isolated upper trapezius transfer especially in the presence of rotator cuff dysfunction [48]. Elhassan reported an average gain in shoulder abduction of 40 degrees post dual upper and lower trapezius transfers with increased shoulder stability and improved average DASH score from 59 preoperatively to 47 postoperatively [30]. In a similar context, Bertelli et al. conducted a combined transfer of the upper trapezius to the proximal humerus and lower trapezius to infraspinatus and reported an average recovered shoulder abduction of 38 degrees [31].

 In the present study, a comparison of mean shoulder abduction postoperative gain was made between those patients who underwent upper trapezius versus combined upper and lower trapezius transfers. The mean gain was very similar, with no statistically nor clinically significant difference.

Free Muscle Transfers

Terzis et al. (2011) transferred different free muscles, including the dual gracilis, adductor longus, and contralateral latissimus dorsi [9]. Terzis et al. (2011) utilized the gracilis to reconstruct elbow flexion and the adductor longus for shoulder abduction for 18 patients [9]. The gracilis and adductor longus have a common vascular pedicle and can be harvested through the same incision on the medial aspect of the thigh [12]. The adductor longus was anchored from the acromion proximally to the lateral humerus at the level of the deltoid groove distally while the gracilis was anchored between the distal clavicle proximally and radial tuberosity distally. There was an average gain of 30 degrees of shoulder abduction following dual gracilis and adductor free transfer [9]. However, this technique is challenging due to the lack of vessels at the recipient site, which necessitates long vein grafts under proper tension for revascularization in addition to finding suitable donor nerves required for neurotization [9]. Terzis et al. (2011) also transferred the latissimus dorsi muscle as a free muscle for shoulder abduction for four patients only. They attached the free latissimus dorsi between the nuchal line and the spinous processes of the cervical and upper thoracic spine proximally and the insertion of the deltoid distally. The average reported gain shoulder abduction after latissimus dorsi transfer was 35 degrees. It could be argued that the main advantage of latissimus dorsi transfer is to preserve the ipsilateral trapezius transfer for later procedures [9].

Review limitations 

Although the studies included in this review reported enhanced outcomes post muscle transfers around the shoulder, the lack of controlled and randomized studies and small cohort groups are the main limitations to making stronger clinical evidence.

## Conclusions

Traumatic brachial plexus injuries could result in significant upper limb disabilities. Primary nerve reconstruction procedures may be implemented first if the presentation was within six months of the injury depending on the nature of lesions and the available donor options. Muscle transfers especially upper trapezius muscle transfers are promising techniques to restore shoulder abduction and enhance shoulder joint stability in the setting of adult traumatic brachial plexus palsy in case of delayed presentation or when primary reconstruction unsatisfactory. The reported clinical results are encouraging, providing support for continued use in these challenging patient populations. However, more comparative data are required to optimize the outcomes and establish stronger clinical evidence.
